# Poly-γ-glutamic acid induces system tolerance to drought stress by promoting abscisic acid accumulation in *Brassica napus* L.

**DOI:** 10.1038/s41598-019-57190-4

**Published:** 2020-01-14

**Authors:** Zongqi Xu, Junjie Ma, Peng Lei, Qian Wang, Xiaohai Feng, Hong Xu

**Affiliations:** State Key Laboratory of Materials-Oriented Chemical Engineering, Nanjing Tech University, Nanjing, 210009 P.R. China

**Keywords:** Plant physiology, Drought

## Abstract

As a new plant biostimulant, poly-γ-glutamic acid (γ-PGA) may be an effective anti-drought agent that can efficiently alleviate the damage to plants under drought stress. In this study, the effects of γ-PGA on the physiological responses of oilseed rape (*Brassica napus* L.) seedlings under drought stress were investigated using hydroponics. Growth and development of the rape seedlings were significantly inhibited in a polyethylene glycol-simulated drought environment. However, 12 d after application of γ-PGA under drought stress, the fresh weight, chlorophyll content, and relative water content of rape seedlings all markedly increased. Moreover, proline content and antioxidant enzyme activity were all markedly enhanced, and the malondialdehyde content was significantly reduced in rape seedlings treated with γ-PGA. Furthermore, the content of the important anti-drought response hormone, abscisic acid (ABA), as well as the expression levels of the ABA metabolism regulation genes *BnNCED3*, *BnZEP*, and *BnAAO4*, significantly increased. These results indicate that γ-PGA may induce elements of a tolerance system to drought stress by promoting ABA accumulation in *B. Napus*.

## Introduction

Drought stress is currently one of the most destructive abiotic stresses, increasing in intensity in recent decades and causing significant losses in agricultural production^1–3^. Drought is predicted to be the main threat to grain production in the near future^4,5^, therefore, improving plant tolerance to drought stress is critical.

Drought stress may cause photosynthetic decline, inhibition of root elongation, and oxidative damage to cells, negatively influencing plant growth and development^6,7^. Plants use several strategies to reduce damage caused by dry environments, including the accumulation of proline which has been shown to effectively alleviate drought-induced osmotic stress^8^. Moreover, antioxidant enzymes including peroxidase (POD), ascorbate peroxidase (APX), superoxide dismutase (SOD), and catalase (CAT) react directly with reactive oxygen species (ROS) under abiotic stress^9–11^. Lipid peroxidation reflects oxidative damage to plant cells and is typically assessed using the level of malondialdehyde (MDA) as an indicator of drought stress damage^12^.

With the discovery of genes related to drought resistance, studies have increasingly focused on abscisic acid (ABA) signal transduction pathway, reported to be involved in plant drought responses^13^. Some stress-responsive genes, including genes encoding osmotic protection proteins, are induced by ABA^14,15^. In addition, some carbohydrates and secondary metabolites are also regulated by ABA in plants^16^. At present, it is recognized that the biosynthesis of ABA in higher plants is mainly through indirect pathway. Zeaxanthin epoxidase (ZEP), 9-*cis*-epoxycarotenoid dioxygenase (NCED) and Abscisic acid aldehyde oxidase (AAO) are the key regulatory enzymes. ZEP catalyzes the conversion of zeaxanthin to violaxanthin in xanthophyll cycle. NCED enzymescleave the cis-isomers of violaxanthin and neoxanthin to form xanthoxin, which is the precursor of ABA, then xanthoxin will finally be oxidized to ABA by AAO^17^.

Poly-γ-glutamic acid (γ-PGA) is a homopolymer consisting of glutamic acid units connected by γ-amide linkages produced by microbes^18,19^. Given its super-chelating, biodegradable, and environmentally friendly characteristics, γ-PGA has great potential for application in agriculture^20^. γ-PGA was reported to significantly increase the dry weight of cucumber seedlings, especially in environments with low nutrition, indicating the synergistic effect of the fertilizer^21^. Moreover, previous studies have emphasized that biopolymers such as polyamino acids and polysaccharides can promote plant growth and enhance tolerance to drought stress^22^. In previous studies, we reported that growth and nitrogen assimilation of plants were significantly improved by γ-PGA application^20,23,24^. Furthermore, γ-PGA enhanced the tolerance of oilseed rape seedlings to salt and cold stress^25,26^. However, only few studies have assessed the effects of γ-PGA on drought resistance in plants. Determining the effects of γ-PGA on plant resistance to drought stress is necessary to understand how to regulate plant growth, which would aid the development of efficient and environmentally friendly drought resistant agents of γ-PGA.

Oilseed rape (*Brassica napus* L.) is among the major globally produced oil crops whose production and quality are the most affected by drought stress^27^. The objective of the present study was to investigate the effects of γ-PGA on rape seedlings and reveal the mechanism of action. Biomass, proline content, MDA content, and antioxidant enzyme activity were compared under polyethylene glycol (PEG) stress. Subsequently, the expression levels of some of oilseed rape drought-associated genes were also evaluated. Moreover, the mechanisms by which γ-PGA enhanced salt resistance in rape seedlings were also elaborated based on the research findings.

## Results

### Effects of γ-PGA on fresh weight, chlorophyll content, and relative water content of rape seedling shoots under drought stress

The effect of γ-PGA on fresh weight and chlorophyll content of rape seedlings is shown in Table 1. Under normal water condition, γ-PGA improved both the FW and chlorophyll content of rape seedlings, especially after 8 d of treatment, consistent with the results of previous studies^25^. After 12 d of treatment, the FW and chlorophyll content of the PGA group increased by 18.9 and 18.6%, respectively, compared to the control group. Under drought stress, rape seedlings in the PEG group showed 60.6 and 54.6% lower FW and chlorophyll content, respectively, compared to the control group after 12 d of treatment. However, the FW and chlorophyll content of rape seedlings in the PGA + PEG group increased by 43.5 and 33.8%, respectively, compared to the PEG group.

**Table 1:** Effects of γ-PGA on fresh weight and chlorophyll content in rape seedling shoots under non-stress and drought stress, respectively.

Days after treatment	Fresh weight (g)				Chlorophyll content (mg·g^−1^ FW)			
	Control	PGA	PEG	PEG + PGA	Control	PGA	PEG	PEG + PGA
2	0.689 ± 0.045a	0.694 ± 0.048a	0.553 ± 0.037b	0.618 ± 0.039ab	2.216 ± 0.124a	2.249 ± 0.125a	1.831 ± 0.121b	1.993 ± 0.124ab
4	0.717 ± 0.048a	0.783 ± 0.046a	0.482 ± 0.035c	0.593 ± 0.041b	2.332 ± 0.125a	2.484 ± 0.128a	1.743 ± 0.120b	1.858 ± 0.122b
6	0.795 ± 0.052a	0.855 ± 0.049a	0.468 ± 0.039c	0.589 ± 0.044b	2.527 ± 0.123a	2.645 ± 0.127a	1.553 ± 0.119c	1.849 ± 0.124b
8	0.873 ± 0.050b	1.012 ± 0.051a	0.441 ± 0.036d	0.573 ± 0.047c	2.684 ± 0.129a	2.915 ± 0.129a	1.472 ± 0.117c	1.742 ± 0.122b
10	0.954 ± 0.053b	1.127 ± 0.053a	0.426 ± 0.039d	0.567 ± 0.045c	2.803 ± 0.142b	3.225 ± 0.149a	1.385 ± 0.116d	1.737 ± 0.120c
12	1.032 ± 0.055b	1.228 ± 0.057a	0.407 ± 0.040d	0.584 ± 0.048c	2.925 ± 0.133b	3.470 ± 0.156a	1.327 ± 0.116d	1.775 ± 0.120c

Each value represents the mean ± standard deviation. Different lowercase letters of each line indicate significant difference at p < 0.05.

**Figure 1:**
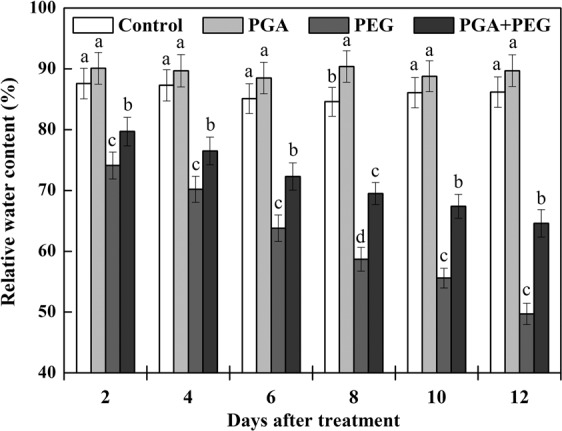
Effects of poly-γ-glutamic acid (γ-PGA) on relative water content in the shoots of rape seedlings under non-stress and drought stress, respectively. Each value represents the mean ± standard deviation (SD). Different lowercase letters indicate significant differences at *p* < 0.05.

The RWC of the rape seedlings remained relatively stable under normal water conditions, and PGA induced a slight increase in the RWC of the rape seedlings, although the increase was not significant (Fig. 1). Under drought stress, the RWC of the oilseed rape seedlings showed a sharp decrease. The RWC decreased by 15.4, 19.6, 25, 30.6, 35.4, and 42.3% after 2, 4, 6, 8, 10, and 12 d of treatment, respectively, compared to the control group. However, the RWC of rape seedlings in the PGA + PEG group increased by 7.6, 9.0, 13.3, 18.4, 21.2, and 30.0% with respect to days after treatment compared to the PEG group. These results indicate that γ-PGA alleviated the effects of drought stress on rape seedlings.

### Effects of γ-PGA on MDA content

**Figure 2:**
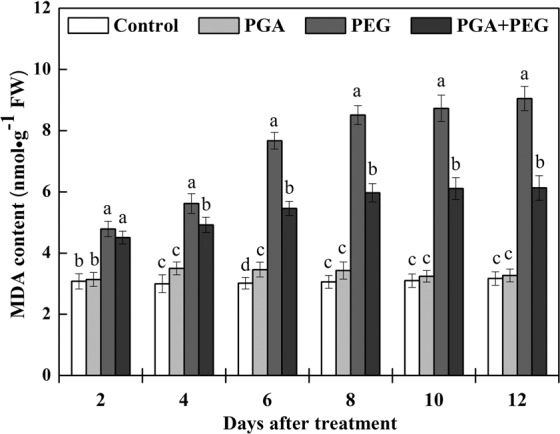
Effects of poly-γ-glutamic acid (γ-PGA) on malondialdehyde (MDA) content in the shoots of rape seedlings under non-stress and drought stress, respectively. Each value represents the mean ± standard deviation (SD). Different lowercase letters indicate significant differences at *p* < 0.05.

Although the MDA content remained at relatively low levels under normal water conditions, it increased in the PGA group, but not significantly (Fig. 2). Under drought stress, the MDA content of rape seedlings in the PEG and PGA + PEG groups increased markedly. However, the PGA + PEG group always showed lower MDA content than the PEG group, especially after 6 d of treatment. After 12 d of treatment, the MDA content in the PGA + PEG group exhibited a 32.3% reduction compared to the PEG group. These results show that γ-PGA effectively alleviated the increase in MDA content of rape seedlings under drought stress.

### Effects of γ-PGA on proline accumulation

**Figure 3:**
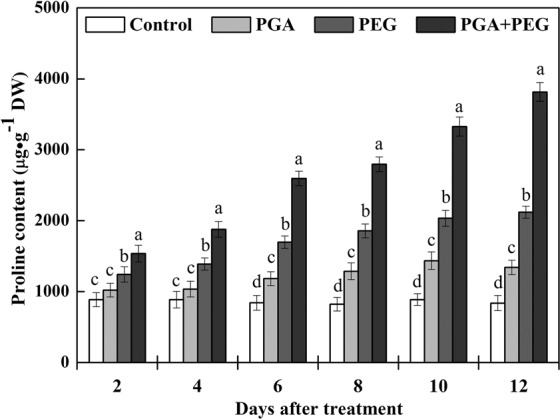
Effects of poly-γ-glutamic acid (γ-PGA) on proline content in the shoots of rape seedlings under non-stress and drought stress, respectively. Each value represents the mean ± standard deviation (SD). Different lowercase letters indicate significant differences at *p* < 0.05.

The proline content in the shoots of the rape seedlings in the PGA group was marginally higher than the control group, especially after 6 d of treatment (Fig. 3). Under drought stress, the proline content of rape seedlings improved significantly in both the PEG and PGA + PEG groups. However, the proline content in the PGA + PEG group was significantly higher than the PEG group, and the trend became more noticeable after 6 d of treatment. After 12 d of treatment, the proline content of rape seedlings in the PGA + PEG group reached 3815.5 µg/g DW, which was 79.9% higher than that in the PEG group. These results show that γ-PGA enhanced the synthesis of proline in rape seedlings, and this enhancement was more pronounced under drought stress.

### Effects of γ-PGA on antioxidant enzyme activity

**Figure 4:**
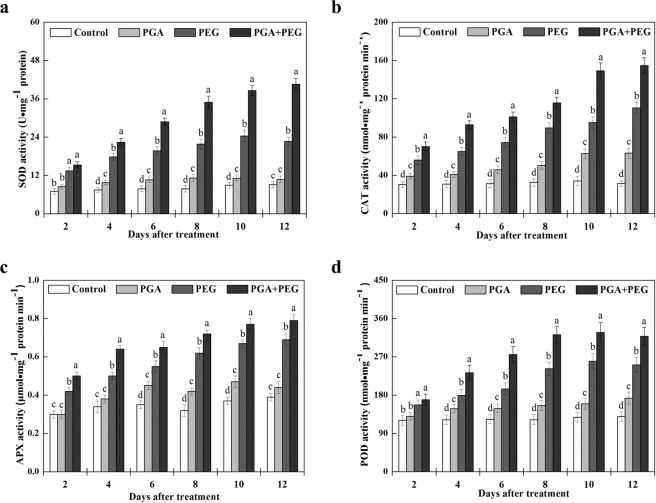
Effects of poly-γ-glutamic acid (γ-PGA) on the superoxide dismutase (SOD) (**a**), catalase (CAT) (**b**), ascorbate peroxidase (APX) (**c**), and peroxidase (POD) (**d**) activities in the shoots of rape seedlings under non-stress and drought stress, respectively. Each value represents the mean ± standard deviation (SD). Different lowercase letters indicate significant differences at *p* < 0.05.

The effect of γ-PGA on the antioxidant enzyme activity of rape seedlings is shown in Fig. 4. The activity changes of SOD, CAT, APX, and POD showed similar trends in our study. SOD, CAT, APX, and POD activities in the PGA group were consistently higher in the PGA group than in the control group. Under drought stress, rape seedling SOD, CAT, APX, and POD activities improved significantly in both the PEG and PGA + PEG groups. In addition, the SOD, CAT, APX, and POD activities in the PGA + PEG group were all significantly higher than those in the PEG group, and always remained at a substantially high level. Consequently, these results show that γ-PGA effectively enhanced the SOD, CAT, APX, and POD activities in rape seedlings, and this enhancement effect was pronounced under drought stress.

### Effects of γ-PGA on ABA accumulation and relative transcription levels of ABA biosynthesis genes

**Figure 5:**
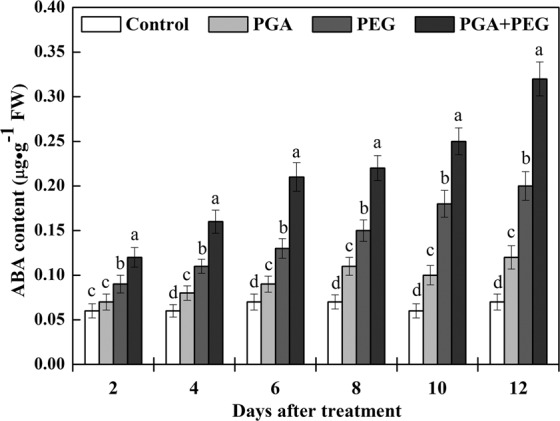
Effects of poly-γ-glutamic acid (γ-PGA) on abscisic acid (ABA) content in the shoots of rape seedlings under non-stress and drought stress, respectively. Each value represents the mean ± standard deviation (SD). Different lowercase letters indicate significant differences at *p* < 0.05.

The effect of γ-PGA on ABA content of rape seedlings is shown in Fig. 5. The ABA content of rape seedlings was slightly higher in the PGA group than in the control group, especially after 4 d of treatment. Under drought stress, γ-PGA application led to a significant increase in ABA content from the beginning, which was 33.3% higher than the PEG group after 2 d of treatment, and this value reached 60% after 12 d of treatment.

**Figure 6:**
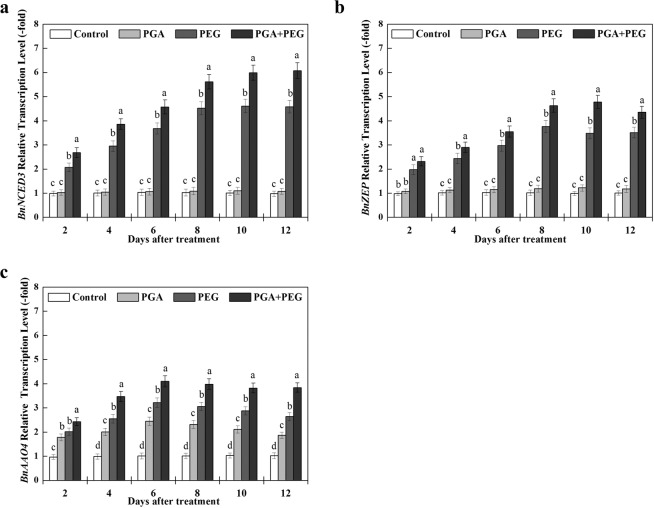
Effects of poly-γ-glutamic acid (γ-PGA) on relative transcription levels of *BnNCED3* (**a**), *BnZEP* (**b**), and *BnAAO4* (**c**) in the shoots of rape seedlings under non-stress and drought stress, respectively. Each value represents the mean ± standard deviation (SD). Different lowercase letters indicate significant differences at *p* < 0.05.

The effects of γ-PGA on the relative transcription levels of *BnNCED3*, *BnZEP*, and *BnAAO4* are shown in Fig. 6. In our study, the transcription levels of *BnNCED3* and *BnZ*EP showed no significant difference between the control and PGA groups, and their transcription could be induced under drought stress. In addition, the application of γ-PGA significantly increased the transcription levels of *BnNCED3, BnZ*EP, and *BnAAO4* under drought stress, and the transcription of these three genes remained at a higher level during the entire sampling time compared with the PEG group. In contrast to *BnNCED3* and *BnZ*EP, the transcription of *BnAAO4* was also induced by γ-PGA under non-stress condition compared to the control group. In general, these results indicate that application of γ-PGA can induce the transcription of specific ABA biosynthesis genes, especially under drought stress, which are associated with ABA accumulation.

## Discussion

As a natural polymeric amino acid, γ-PGA plays an important role in agricultural applications, as has been extensively reported^20^. However, there are few reports on the effects of γ-PGA on drought resistance in plants. This paper is the first to report the effects and mechanism of action of γ-PGA in drought stress tolerance. As shown in Table 1, γ-PGA increased the FW and chlorophyll content of rape seedlings under non-stress conditions, consistent with our previous results^25^. In addition, and as shown in Fig. 1, the application of γ-PGA led to a marginal increase in the RWC of rape seedlings under non-stress conditions, indicating that γ-PGA can enhance plant water holding capacity. Furthermore, γ-PGA application effectively reduced water loss in rape seedlings and inhibited the sharp decrease in RWC under drought stress.

To further investigate the mechanisms of γ-PGA enhanced drought stress tolerance in rape seedlings, we investigated the physiological indexes of rape seedlings under drought stress. Drought stress caused a significant increase in MDA content in rape seedlings, with the change in MDA levels reflecting plant cytoplasmic membrane peroxidation under stress^28^. Drought stress can reduce the utilization of oxygen in plants, resulting in excess ROS such as O^2−^ and H_2_O_2_ and leading to cell membrane damage and affecting plant growth^29^.

In this study, γ-PGA significantly inhibited the increase in MDA levels in rape seedlings under drought stress. This result indicates that γ-PGA can effectively promote the removal of excessive ROS produced in rape seedlings in adverse environments, reducing the production of MDA and consequently the damage to rape seedlings. We also investigated the antioxidant activity of four enzymes important for plant defense against ROS, namely, POD, APX, SOD, and CAT. In plant cells, POD catalyzes the conversion of H_2_O_2_ to H_2_O and O_2_, SOD and APX catalyze the disproportionation of O^2−^ into molecular O^2^ and H_2_O_2_, and CAT activity leads to the elimination of H_2_O_2_ produced by β-oxidation of fatty acids^30–33^. The activity of these enzymes is normally upregulated when plants are subjected to external stress, scavenging excess free radicals and maintaining free radicals at physiological dynamic levels to enhance plant resistance. Under normal water conditions, γ-PGA application resulted in a relative increase in POD, APX, SOD, and CAT activities in rape seedlings, and the effect of γ-PGA on enzyme activity was more significant, and maintained at a higher level, under drought stress. Therefore, γ-PGA can effectively increase the POD, APX, SOD, and CAT activities under drought stress, thereby enhancing the ability of plants to scavenge ROS and ultimately reduce MDA-induced damage. In addition, osmotic pressure in plants is altered under drought stress, so there must be related substances to maintain the stability of osmotic pressure. Proline is the most important osmotic regulator, which is widely present in plants in a free state^34^. Studies have shown that proline accumulation is positively correlated with plant adaptation to drought and salt stress^35^. In this study, γ-PGA increased the proline content in rape seedlings under non-stress, and the improvement was more significant under drought stress.

Plant drought stress-related signal transduction pathways are interconnected and interact to form a signal transduction network, of which ABA is an important component^36^. Studies have shown that an increase in ABA content can prevent the reduction of antioxidant enzyme activity in plants, regulate the balance of active oxygen metabolism, and inhibit the increase in MDA content, thereby mitigating drought damage^37^. Moreover, ABA can activate the expression of drought-tolerance related genes^22^. Cutler *et al*. and Lee *et al*. both observed that ABA can significantly decrease plant leaf elongation and promote stomatal closure, resulting in reduced leaf area, reduced water loss, and improved water use efficiency under drought stress^38,39^. Jie *et al*. found that the application of ABA can increase proline content, thereby protecting apple leaves from oxidative damage induced by drought stress^40^. Sharp *et al*. showed that the addition of exogenous ABA promoted tomato plant growth and increased water uptake^41^. Therefore, we speculated that γ-PGA application might reduce water loss and ease oxidative damage in rape seedlings under drought stress in a manner related to increased ABA accumulation.

The ABA content increases rapidly when plants are subjected to drought stress, and this effect is achieved mainly via the activation of ABA biosynthesis and inhibition of the ABA degradation pathway^42,43^. To test this, we first investigated the effect of γ-PGA on the accumulation of ABA. γ-PGA promoted the accumulation of ABA in rape seedlings even under non-stress conditions, and ABA accumulation was greater under drought stress.

In *B*. *Napus*, *BnZEP* gene has high homology with *Arabidopsis thaliana AtZEP* gene^44^. Hee *et al*. showed that the overexpression of *ZEP* gene in Arabidopsis can improve its ability to resist osmotic stress^45^. NCEDs are members of a multigene family found in manyplant species. Xu *et al*. found the overexpression of the *B. napus* NCED family gene *BnNCED3* contributed to ABA accumulation in transgenic *Arabidopsis* plants, thereby enhancing abiotic stress tolerance^46^. Among four *AAO*s (*AAO1*-*AAO4*), *AAO4* gene is the most likely candidate for an ABA biosynthetic enzyme because in addition to its high expression in developing siliques, it is also induced by drought stress in leaves^47^. To further confirm that γ-PGA promoted ABA accumulation, we investigated the transcription levels of *BnNCED3*, *BnZEP*, and *BnAAO4*. In this study, γ-PGA effectively increased the transcription levels of these three genes under drought stress, and *BnAAO4* was also induced by γ-PGA application under non-stress conditions. These results indicate that γ-PGA may induced ABA accumulation by upregulating the transcription levels of *BnNCED3*, *BnZEP*, and *BnAAO4*.

An increasing number of plant peptide hormones have recently been identified, and their roles in plant growth, development, and stress responses have gradually been revealed^48^. However, it remains unclear how γ-PGA regulates ABA synthesis and expression of related ABA biosynthesis genes; in addition, the signal transduction mechanism downstream of γ-PGA in plants needs further clarification, although it was shown in a previous study that γ-PGA can trigger a change in calcium signaling, promoting growth in the Chinese cabbage^23^.

**Figure 7:**
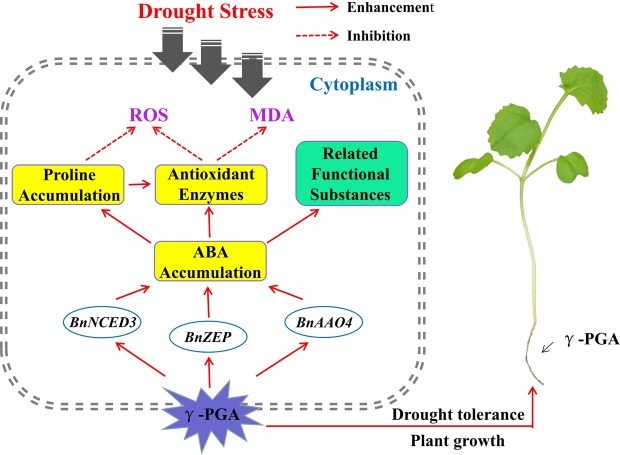
Possible mechanism of γ-PGA-induced system tolerance to drought stress in *Brassica napus* L. The solid arrows represent enhancement, whereas the dotted arrows represent inhibition.

The possible mechanism of γ-PGA induced system tolerance to drought stress in *B*. *napus* are shown in Fig. 7. In summary, the application of γ-PGA activates ABA-associated regulatory factors, thereby up-regulating the expression of ABA biosynthesis genes and promoting the accumulation of ABA. ABA further regulates downstream targets, increasing the activity of antioxidant enzymes and promoting the accumulation of proline; this increases cellular ROS scavenging ability and osmoregulation in rape seedlings, reducing protein damage and membrane lipid peroxidation resulting from drought stress. However, further experimental evidence is required to confirm these observations.

In summary, our study showed the positive effects of γ-PGA on rape seedlings under drought stress. This is mainly reflected in increased biomass and proline content, improved water retention capacity, reduced MDA content, and enhanced activity of antioxidant enzymes. Moreover, ABA accumulation was related to increased transcription levels of the ABA biosynthesis genes *BnNCED3*, *BnZEP*, and *BnAAO4*. Our results indicate that γ-PGA induced system tolerance to drought stress by promoting ABA accumulation in *B*. *napus*.

## Materials and Methods

### Plant materials and stress treatments

The experiments were carried out with Suyou No. 1 (*B*. *napus*), provided by Jiangsu Academy of Agricultural Sciences, Nanjing, China. The seeds were germinated on filter paper soaked in distilled water for 5 d. Seedlings were then transplanted to hydroponic grow plastic boxes and were grown in growth chambers with supplemental lighting of 250 µmol m^−2^ s^−1^, a 14/10 h day/night photoperiod, relative humidity of 65%, and a controlled temperature of 24/18 °C (day/night). The nutrient solution was based on Hoagland Arnon’s nutritive solution with minor modifications^25^. Morphologically uniform four-week-old seedlings were selected for different treatments.

Seedlings were randomly divided into four groups with three replicates each. We simulated drought stress by adding PEG-6000 (150 g/L) to the hydroponic solution, the corresponding osmotic potential of the solution was about −0.40 MPa^49^. The four groups were cultured in half-strength modified Hoagland’s solution containing different components, as follows: Control, Hoagland’s solution only; PGA, Hoagland’s solution with PGA (20 mg/L, 2 kDa); PEG, Hoagland’s solution with PEG-6000 (150 g/L); and PGA + PEG, Hoagland’s solution with PGA (20 mg/L, 2 kDa) and PEG-6000 (150 g/L). The concentration and molecular weight of γ-PGA were selected based on our previous study^25^; γ-PGA was provided by Nanjing Shineking Biotechnology Co. Ltd. The hydroponic solution was replenished daily, and seedlings were sampled at 2, 4, 6, 8, 10, and 12 d after treatment.

### Determination of fresh weight and relative water content

Six plants per replicate were harvested and weighed immediately after removing the roots. The determination of relative water content (RWC) is based on the method of Smart^50^, fresh shoots were weighed quickly to obtain fresh weight (FW); the shoots were then soaked in distilled water for 4 h and turgid weight (TW) was measured. To measure dry weight (DW), the shoots were then dried at 80 °C for 24 h. RWC was calculated as follows: RWC (%) = 100% × (FW–DW)/(TW–DW).

### Determination of chlorophyll content

Chlorophyll content was determined following the method described by Arnon^51^. Fresh leaves, each containing 0.5 g of plant tissue, were extracted in the dark with 10 mL of 80% acetone. The assay mixture was centrifuged at 10000 × *g* for 5 min, and the supernatant was removed, mixed with 85% aqueous acetone solution to an appropriate concentration, and the absorbance measured at 663 and 645 nm. Chlorophyll content was calculated according to the equations given by Arnon^51^.

### Measurement of MDA

MDA levels were determined by reaction with 2-thiobarbituric acid according to Cheng *et al*.^52^, with modifications. Briefly, fresh seedling shoot samples (0.3 g each) were homogenized and extracted in 10 mL of 0.25% TBA made in 10% trichloroacetic acid. The mixture was then heated in a water bath shaker at 95 °C for 30 min and rapidly cooled on ice. The absorbance of the supernatants was determined at 532 nm after centrifugation at 5000 × *g* for 10 min. Correction of non-specific absorption was calculated by subtracting the absorbance at 600 nm. The concentration of MDA was expressed as μmol/g FW.

### Determination of proline content

Proline content was measured using the method described by Bates *et al*.^53^, with modifications. 0.5 g seedling shoots samples were ground in liquid nitrogen with a mortar and pestle, 10 mL of 3% sulfosalicylic acid was subsequently added, and the solution was then homogenized in a water bath at 100 °C. The supernatant was collected after centrifugation at 12000 × *g* for 10 min. Subsequently, 2 mL of glacial acetic acid and 3 mL of 2.5% ninhydrin reagent were added to 2 mL of supernatant. The mixture was incubated in boiling water for 60 min, and the reaction mixture was then extracted by adding 4 mL of methylbenzene. The absorbance of the extract liquor was determined at 520 nm, using a spectrophotometer.

### Determination of antioxidant enzymes

For the analysis of antioxidant enzymes (SOD, CAT, APX, and POD), 0.5 g of seedling shoots was manually ground with a mortar and pestle in liquid nitrogen and homogenized in 8 mL of cold extraction buffer consisting of 50 mM sodium phosphate buffer with 1% polyvinylpyrrolidone (pH 7.8). The homogenate was centrifuged at 10000 × *g* for 10 min at 4 °C and the supernatant was used for the measurement of enzyme activity, as described below^54^.

SOD (EC 1.15.1.1) activity was determined by inhibiting photochemical reduction using nitro blue tetrazolium (NBT)^55^. Briefly, 100 μL of enzyme extract was added to the reaction mixture contain ning 50 mM potassium phosphate buffer (pH 7.8), 2 μM riboflavin, 75 μM NBT, 13 mM methionine, and 0.1 mM EDTA. The reaction was started and maintained with 4000 lux light for 20 min. One unit of SOD activity was defined as the amount of enzyme required for 50% inhibition of NBT reduction measured at the wavelength of 560 nm. CAT (EC 1.11.1.6) activity was determined as follows: 100 μL of enzyme extract was added to a 3 mL reaction mixture containing 50 mM potassium phosphate buffer (pH 7.0), 10 mM H_2_O_2_, and 2 mM EDTA-Na_2_. The absorbance changes of the reaction mixture were recorded at 240 nm for 1 min, and CAT activity was expressed as the amount of decomposed H_2_O_2_^56^. The APX (EC 1.11.1.11) activity was measured in a 3 mL reaction mixture consisting of 100 μL of enzyme extract, 100 mM phosphate (pH 7.0), 0.3 mM ascorbic acid, 0.06 mM H_2_O_2_, and 0.1 mM EDTA-Na_2_. The change in absorption was monitored for 30 s at 290 nm after adding H_2_O_2_^57^. The POD (EC1.11.1.7) activity was assayed according to Liu *et al*.^22^, with some modifications. The reaction system contained 50 mM potassium phosphate buffer (pH 7.8), 200 mmol/L H_2_O_2_, 25 mmol/L guaiacol, and 100 μL of enzyme extract. The POD activity was measured by absorbance change recorded at 470 nm.

### Determination of ABA content

ABA content was measured using the methods described by Liu *et al*.^58^. Oilseed rape seedling shoots (1 g) were freeze-dried, then quickly ground into powder and 2.5 mL of extraction buffer (90% methanol, 200 mg/L sodium diethyldithiocarbamate trihydrate) was added for 10 min, with shaking. The extracts were transfered to a covered, siliconized borosilicate tube and incubated overnight in darkness at 4 °C. The extracts were centrifuged at 8000 × *g* for 5 min and vacuum centrifuged at 4 °C to evaporate the supernantant. The residue was dissolved with 500 µL methanolic Tris buffer (10% methanol, 50 mM Tris, 1 mM MgCl_2_, 150 mM NaCl). The ABA content was measured with ELISA kit (code JM-01148P2, Jingmei Bio Inc,. Jiangsu, China) according the manufacturer’s.

### Total RNA extraction and real-time quantitative reverse transcription PCR (qRT-PCR)

Seedling shoots from different treatment groups were sampled at 2, 4, 6, 8, 10, and 12 d for qRT-PCR. Total RNA was isolated using the RNAiso Plus Kit (code 9108, TaKaRa Bio Inc., Shiga, Japan). The PrimeScript RT Master Mix Kit (code RR036A, TaKaRa Bio Inc., Shiga, Japan) was used for reverse transcription of RNA. An ABI StepOnePlus System (Applied Biosystems) was used for qRT-PCR according to the SYBR® *Premix Ex Taq*™ II (Code RR820A; TaKaRa Bio Inc., Shiga, Japan) protocol. The reaction mixture contained 2.0 µL of cDNA, 10 µL of 2 × SYBR® *Premix Ex Taq*™ II, 0.4 µL of 50 × ROX Reference Dye, 0.8 µL of 10 mM of each primer, and 6.0 µL of sterilized distilled water. The qRT-PCR conditions were: 95 °C for 30 s, followed by 40 cycles of 95 °C for 5 s, 60 °C for 30 s, 72 °C for 30 s. The sequences of the specific primers used in this study are shown in Table 2, and the primers were designed and synthesized by GenScript (Nanjing) Co. Ltd. Gene expression patterns were calculated by the 2^−∆∆CT^ method^59^.

**Table 2:** Primer sequences for qRT-PCR.

Gene (ID)	Encoded protein	Primer sequence
*Actin* (AF111812)	Reference gene	L1-5′ AAGAGCTGGAGACGGCTAAG 3′
		R1-5′ GTACTTCAGGGCAACGGAAT 3′
*BnNCED3* (HQ260434)	9-*cis*-Epoxycarotenoid dioxygenase	L1-5′ GGTTCCGTATGGGTTTCACG 3′
		R1-5′ TATGCACACACCATCCCACT 3′
*BnZEP* (GU361616)	Zeaxanthin epoxidase	L1-5′ CGAACACGGGACCTATCTCA 3′
		R1-5′ TGGGAGTTGTCCTGATCACC 3′
*BnAAO4* (EV088287)	Aldehyde oxidase	L1-5′ GCAGTTAGGCTTTGCTGTGT 3′
		R1-5′ GAACGAGCTGACAAGTCCAC 3′

### Statistical analysis

Data are expressed as the mean ± standard deviation. SPSS 17.0 software was used for statistical analysis using a one-way ANOVA followed by Duncan’s test (*p* < 0.05).
